# Supplementary material for: miR-210 Regulates Apoptotic Cell Death during Cellular Hypoxia and Reoxygenation in a Diametrically Opposite Manner
Source: Biomedicines. 2021 Dec 25;10(1):42. doi: 10.3390/biomedicines10010042 (PMC8772724; doi:10.3390/biomedicines10010042)
Supplement: Supplementary file 1 [file biomedicines-10-00042-s001.zip › biomedicines-1478683-supplementary.pdf]

Supplementary Data and Materials

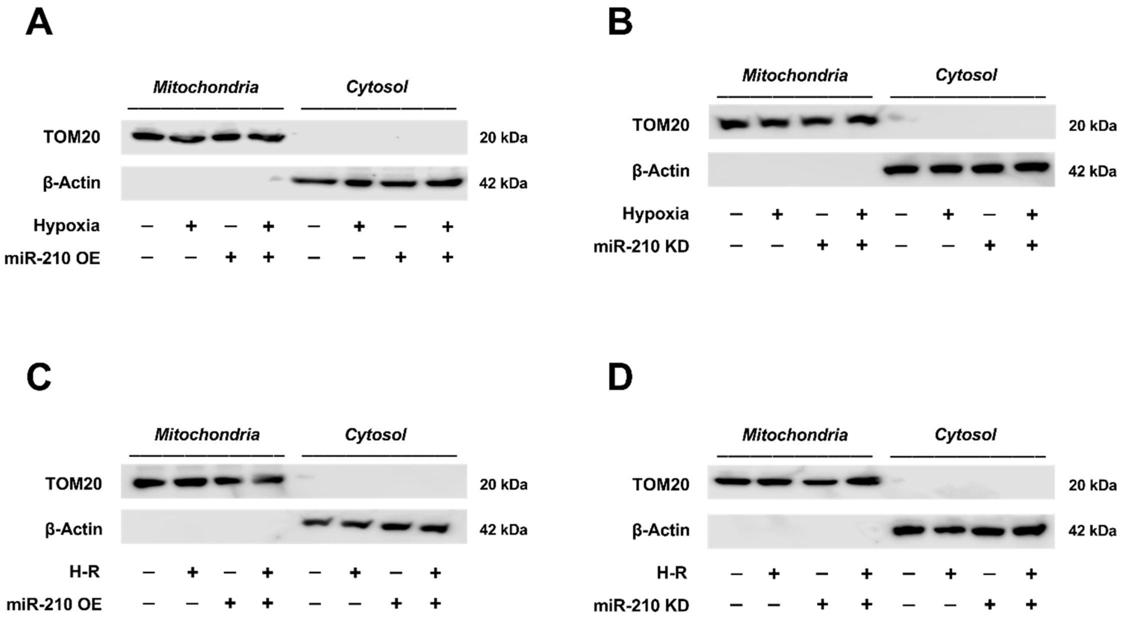

Supplementary Figure S1: Western blot analysis determining the validity and integrity of the *mitochondrial* and *cytosolic* fractions used for the *cytochrome c* ELISA assay. (A-D) Representative Western blots demonstrate the validity and integrity of the segregated *mitochondrial* and *cytosolic* fractions. TOM20 was used as a marker for the *mitochondrial* fractions while β-actin was used as a marker for the *cytosolic* fractions. The *loading inputs* for the *mitochondrial* fractions and the *cytosolic* fractions were set at 5 µg of protein equivalent and 20µg of protein equivalent, respectively. The origin, source, and the respective used amounts of the TOM20 and β-actin antibodies is depicted in Table 2.

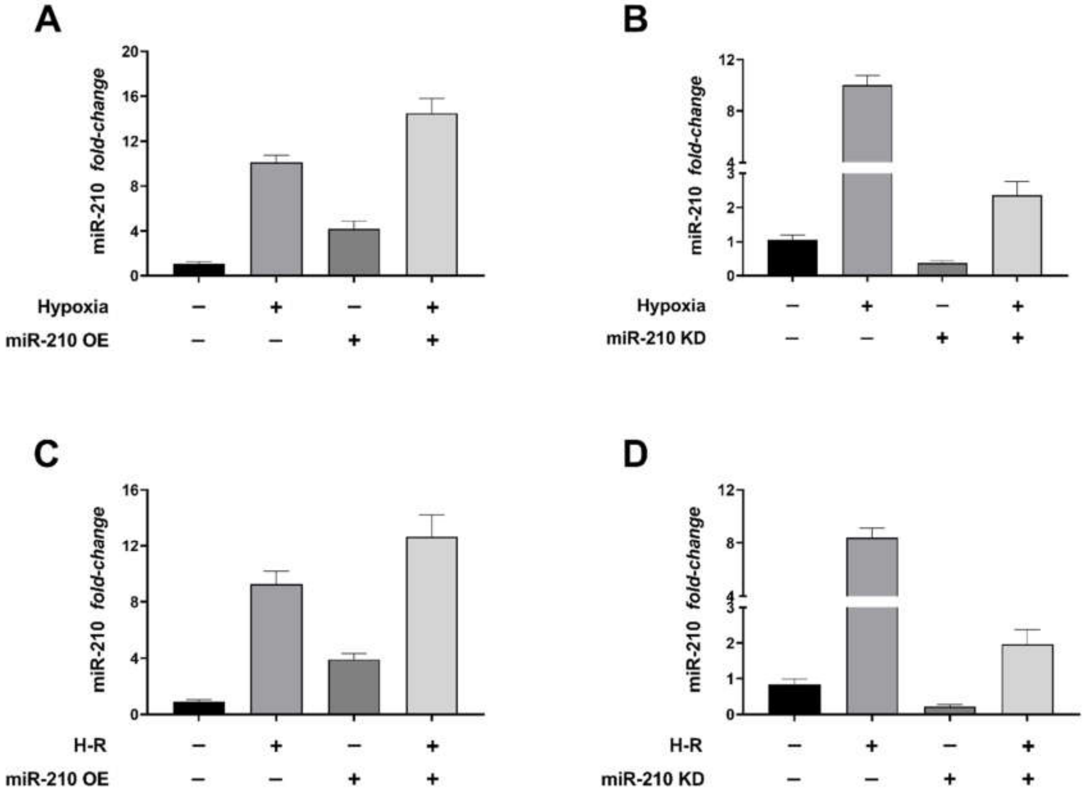

Supplementary Figure S2: miR-210 hybridization immunoassay validating miR-210 overexpression and miR-210 knockdown in the respective *input* lysates dedicated for the *Apoptosome complex formation* assay. (A-D) Quantitative miR-210 hybridization immunoassays unequivocally validate the overexpression and knockdown of miR-210 in the respective experimental groups. miR-210 expression levels are depicted as *fold-change ± S.D* from three (3) technical replicates for each of the four biological replicates belonging to each experimental group (n=4). OE: miR-210 overexpression; KD: miR-210-3p *decoy/inhibitor*; H-R: hypoxia-reoxygenation; S.D: standard deviation

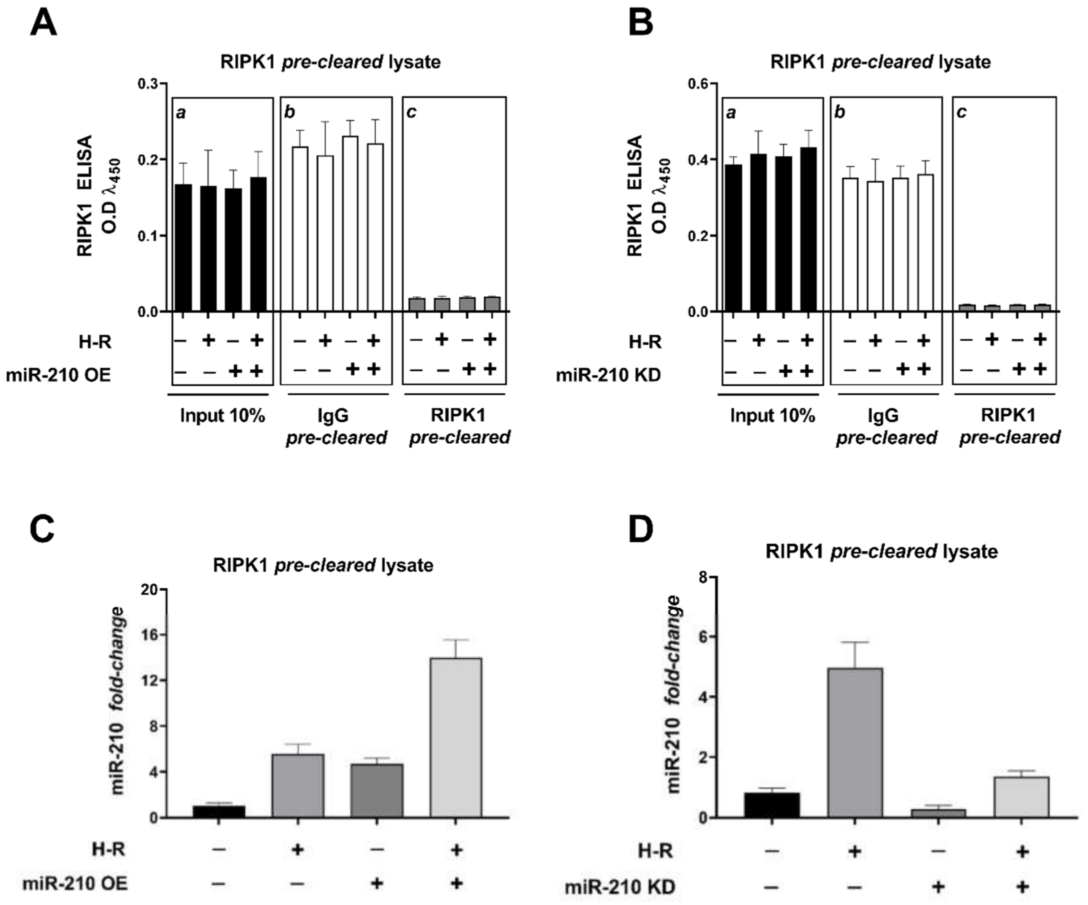

Supplementary Figure S3: ELISA immunoassays validating the integrity of the *RIPK1-precleared lysates* subjected to FADD immunoprecipitation for the *DISC-IIa Complex analysis*. (A,B) Quantitative ELISA immunoassays show the relative depletion of RIPK1 in the *RIPK1-precleared lysates* (A *inset c* and B *inset c*) compared to the Rabbit IgG precleared lysates (A *inset b* and B *inset b*) and the *inputs* of native lysates (A *inset a* and B *inset a*). Data is expressed as experimental *blank-corrected* absorbances measured at λ_{450} (450 nm). (C,D) miR-210 expression level in the *RIPK1-precleared lysates* was determined by the miR-210 hybridization immunoassay, as described in the Methods. Data is expressed as *Mean ± SD* from three (3) technical replicates for each of the four biological replicates belonging to each experimental group (n=4). miR-210 expression levels are depicted as *fold-change ± S.D*

OE: miR-210 overexpression; KD: miR-210-3p *decoy/inhibitor*; H-R: hypoxia-reoxygenation; S.D: standard deviation

Supplementary Table S1: Composition of the *hypoxia medium*

Component	500 mL	Final concentration	Source

			(Notation)
DMEM, No Glucose	464.45 mL	93% v/v	1
Creatine	131.2 mg	5 mM	2
D-(+)-Glucose Solution 2.5 M, 450 g/L	0.55 mL	2.75 mM	3
Glutamine 200 mM	5 mL	2 mM	4
HEPES 1M	5 mL	10 mM	5
L-Carnitine, 200 mM	5 mL	2 mM	6
Non-essential Amino Acids, 100x	5 mL	N/A*	7
Sodium Pyruvate 100 mM	5 mL	1 mM	8
Taurine 500 mM	5 mL	5 mM	9
Linoleic Acid-Oleic Acid-Albumin, 100x	5 mL	N/A*	10

Supplementary Table S1: Notation legend

[1] Thermo Fisher Scientific, Oslo, Norway, Catalogue # 11966025
[2] Sigma Aldrich / Merck Millipore / Merck Life Science, Darmstadt, Germany, Catalogue # C3630-100G
[3] Sigma Aldrich / Merck Millipore / Merck Life Science, Oslo, Norway, Catalogue # G8769
[4] Thermo Fisher Scientific, Oslo, Norway, Catalogue # A2916801
[5] Sigma Aldrich / Merck Millipore / Merck Life Science, Darmstadt, Germany, Catalogue # H4034-500G
[6] Sigma Aldrich / Merck Millipore / Merck Life Science, Darmstadt, Germany, Catalogue # C0283-25G
[7] Thermo Fisher Scientific, Oslo, Norway, Catalogue # 11140035
[8] Thermo Fisher Scientific, Oslo, Norway, Catalogue # 11360070
[9] Sigma Aldrich / Merck Millipore / Merck Life Science, Darmstadt, Germany, Catalogue # T8691-100G
[10] Sigma Aldrich / Merck Millipore / Merck Life Science, Darmstadt, Germany, Catalogue # L9655-5ML
* N/A - Not Applicable
